# Comparison of surgical correction rates between titanium and cobalt-chrome-alloy as rod materials in adolescent idiopathic scoliosis

**DOI:** 10.1038/s41598-020-66975-x

**Published:** 2020-06-22

**Authors:** Jae Hyuk Yang, Seung Woo Suh, Dong-Gune Chang

**Affiliations:** 1 Department of Orthopaedic Surgery, Korea University Guro Hospital, Guro-Gu, 08308 Seoul Republic of Korea; 2 Department of Orthopaedic Surgery, Inje University Sanggye Paik Hospital, College of Medicine, Inje University, 1342, Dongil-Ro, Nowon-Gu, Seoul 01757 Republic of Korea

**Keywords:** Neurosurgery, Neuromuscular disease

## Abstract

Numerous biomechanical studies comparing titanium (Ti) and cobalt-chrome-alloy (CCM) rods are described in the literature. However, there is a dearth of literature comparing the two rod materials in adolescent idiopathic scoliosis (AIS). Therefore, the purpose of this study is to compare the correction rates of Ti and CCM rods in the treatment of AIS with double major curves. We enrolled 45 patients with AIS who underwent surgery between 2009 and 2012. We divided patients into two groups, Group A (n = 29) treated with six-millimeter Ti rods and Group B (n = 16) treated with six-millimeter CCM rods. The rod-derotation maneuver was used for correction. We measured pre- and postoperative indices of coronal alignment (Cobb’s angle, coronal balance, T1-tilt, clavicle angle) and sagittal alignment (sagittal vertical axis, thoracic kyphosis, lumbar lordosis). In our study, there were no significant differences between the two groups with respect to demographics or curve characteristics (*P* > 0.05). In Group A, thoracic and lumbar curvature correction rates were 71.2% and 66.8% respectively, and in Group B they were 71.2% and 73.3%, respectively (*P* = 0.664 and 0.09). There were no significant differences between the two groups in coronal or sagittal factors (*P* > 0.05) except for greater postoperative lumbar lordosis in the CCM group (*P* < 0.001). In conclusion, Ti and CCM rods showed similar correction rates in the sagittal and coronal planes for the surgical correction of AIS with double major curves. Biomechanical studies of Ti and CCM rods *in vitro* is different in biological condition.

## Introduction

The goals of surgery for adolescent idiopathic scoliosis (AIS) are to correct the deformity and achieve sagittal and coronal balance with fusion of the least number of vertebral segments^1^. These goals are affected by factors including patient characteristics (e.g., curve pattern and flexibility) and surgical variables such as the type of device used (hooks or pedicle screws), rod type, and technique used to achieve curve correction (translation, rod-derotation, or direct vertebral rotation)^2–6^. Of these two considerations, surgical variables can be controlled and thus are modifiable. The medical community has been striving continuously to improve available instrumentation to yield better, safer, and stronger implants.

The pedicle screw-rod system has proven its efficiency due to a strong pull-out force and three-column fixation, and it has become the state-of-art technique for posterior spinal fixation^7,8^. Pedicle screw-rod constructs using titanium-alloy (Ti) have become popular in recent years. Ti rods have distinct advantages over their stainless steel predecessors in aspects of more flexibility, a higher inert nature, biocompatibility, corrosion resistance, and an apparent ability to integrate with the surrounding bone with lesser radiologic artifacts^9^. Despite their widespread use, there are concerns about Ti rod implant failure. Ti rods are subjected to weakening of forces during intra-operative rod contouring and corrective procedures in deformity correction surgeries.

As the pursuit of the ideal biomaterial continues, use of cobalt-chrome-alloy (CCM) rods has been on the rise. CCM rods have the merits of both Ti and stainless steel rods, but CCM has higher rigidity than Ti making it possible to use a lower-profile rod with similar strength. Other proposed advantages include relatively low resistance to infection and radiological artifact not significantly different from titanium^9–11^. However, the benefits of CCM over Ti have only been studied *in vitro* biomechanical studies so far, with rare clinical studies comparing their correction rates *in vivo*. With this background, our study was designed to compare and analyze the efficacy of CCM and Ti rod systems with regard to the correction rates of scoliotic curvature in AIS patients with double major curves requiring fusion surgery.

## Material and methods

This research was designed as prospective, consecutive, non-randomized study and patients who underwent surgery for scoliosis between 2009 and 2012 were included. Inclusion criteria were: (1) 11 to 17 years old, (2) Risser grade 1 to 4, (3) scoliosis deformity with a double curve defined as Lenke type 6 which has thoracic and lumbar structure curve more than 30 degrees which also satisfying Suk’s guidelines for fusion as a double major curve (4) a minimum follow-up period of 5 years^6,12^. Patients undergoing revision spine surgery were excluded. This study was performed after obtaining approval of the institutional review board. All methods were carried out in accordance with relevant guidelines and regulations of our licensing committee. Informed consent for surgery and publication of identifying images were obtained and approved from both all patients and parents and/or legal guardians.

To avoid bias of surgical technique cases operated by a single surgeon were enrolled. Posterior spinal instrumentation and fusion were performed using mono-directional pedicle screws. The proximal level of fusion was neutral vertebrae and distal extent of fusion was determined according to Suk’s guidelines^6,12^. In Group A, 6-mm Ti rods (Titanium Alloy Ti 6AI-4V, Carpenter, PA, USA) was used and in Group B, 6-millimeter CCM rods (BioDur^®^ Carpenter CCM^®^ Alloy, Carpenter, PA, USA) was used. Correction was obtained with a simultaneous double rod-derotation maneuver. Thoracoplasty was performed in patients who had a significant rib hump height difference of more than 3 cm. Radiographs were taken at regularly with interval of 6 months, and final radiographs taken at last follow-up visit were compared with the preoperative radiographs.

Coronal Cobb’s angles, coronal balance (CB), first thoracic vertebral tilt (T1 angle), and clavicle angle (CA) were assessed to evaluate coronal balance^13^. Sagittal vertical axis (SVA), thoracic kyphosis (TK) and lumbar lordosis (LL) were assessed to evaluate sagittal balance^14^. Normal values based on the Korean sagittal profile as elaborated by Lee and colleagues were used as a ref. ^15^. For verifying inter and intra-observer reliability, all factors were measured twice by experienced orthopaedic surgeons and radiologists. After the first measurement, a second measurement was performed with a three-week of time-interval.

Clinical outcomes related with complication were recorded as form of intra-operative, immediate post-operative and last follow-up. As intra-operative complications, significant change of neuromonitoring signal and screw pull out during correction procedure were evaluated. As immediate complications, infection, pulmonary complication (hemothorax or pneumonia etc.), wound dehiscence, neurological deficit, abdominal discomfort were evaluated. And as late complications, delayed infection, reduction loss which was defined as change of angle more than 5 degrees, proximal and distal decompensation, halo sign or screw breakage on last follow-up radiography, kidney and/or liver complication related with metal debris were evaluated.

Statistical analysis was performed using SPSS Version 17.0 (IBM Corporation, Armonk, NY, USA). Pearson’s chi-square test or Fisher’s exact test was used to compare ordinal values between the two groups. The Mann–Whitney U test was used to compare numeric values between the two groups. If the P value is under 0.05, this value was considered to be statistically significant. However, because the enrolled patients for statistical analysis are relatively small in size, the power of statistically significant result was verified with G-power program Ver 3.19 (Universität Düsseldorf, Germany). Intra and inter-observer reliability were analyzed with intraclass correlation coefficient (ICC) test.

## Results

Group A included 29 patients who underwent correction with Ti rods and Group B consisted of 16 patients who underwent correction with CCM rods for a total study population of 45 patients. Before comparison of two groups, intra and inter reliability of all measured factors were verified and all values were more than 0.9 (Table 1).

**Table 1:** Result of intra and inter-observer reliability test using intra-class coefficient test.

Factors		Intra-observer reliability	Inter-observer reliability
Cobb’s angle (°)	Pre-operative	0.99	0.99
	Post-operative	0.94	0.94
Coronal balance (mm)	Pre-operative	0.98	0.98
	Post-operative	0.98	0.98
T1 tilt angle (°)	Pre-operative	0.98	0.97
	Post-operative	0.98	0.96
Clavicle angle (°)	Pre-operative	0.92	0.91
	Post-operative	0.94	0.93
Thoracic kyphosis (°)	Pre-operative	0.99	0.99
	Post-operative	0.98	0.98
Lumbar lordosis (°)	Pre-operative	0.99	0.99
	Post-operative	0.98	0.97
Sagittal balance (mm)	Pre-operative	0.98	0.98
	Post-operative	0.99	0.99

Both groups were matched for possible confounding factors that could affect the correction rate^1,14^. There was no significant difference in age, sex, height, weight, body mass index, Risser stage, preoperative Cobb’s angle, flexibility of major curve, Lenke curve type, fusion length, upper and lower fusion level or thoracoplasty between the two groups (Table 2). Pedicle screws were inserted in all fusion level, therefore the density of pedicle screws between Ti and CCM groups could be considered to be same. Postoperative complications occurred in 3 and 2 cases in Ti and CCM group, respectively. There was no significant difference in postoperative complications between the two groups (*P* = 0.991) and it was described detail in Table 2. Follow-up period was calculated 7.6 ± 1.8 and 6.0 ± 0.9 years in Group A and B. Late post-operative complications developed 3 cases in Ti group and 0 cases in CCM group (*P* = 0.267).

**Table 2:** Demographic information, surgical characteristics and postoperative complications of the enrolled patients.

Factors	Group A (n = 29) Titanium-alloy rod	Group B (n = 16)Cobalt-chrome-alloy rod	*p*
Age (years)	14.1 ± 1.5	13.9 ± 1.6	0.964*
Sex	Male (2), Female (27)	Male (0), Female (16)	0.541^†^
Height	154.3 ± 8.0	156.6 ± 5.4	0.569*
Weight	45.5 ± 6.4	45.2 ± 8.5	0.250^*^
BMI	19.3 ± 3.4	18.4 ± 3.4	0.158*
Risser stage	1 (2), 2 (3), 3 (3), 4 (21)	1 (4), 2 (2), 3 (3), 4 (7)	0.235^†^
Preoperative Cobb’s Angle (Major curve)	60.1 ± 14.2°	60.1 ± 12.3°	0.729^*^
Flexibility of curve (Major curve)	29.6 ± 11.5%	27.1 ± 10.8%	0.494*
Lenke curve type	6 (29)	6 (19)	0.988^†^
Fusion length	12.2 ± 1.3	12.2 ± 1.2	0.958^†^
Upper Instrumented Vertebrae	2.7 (2–4)	2.9 (2–4)	0.572^†^
T2	10	5	
T3	17	11	
T4	2	3	
Lower Instrumented Vertebrae	3.0 (1–4)	3.3 (2–5)	0.169^†^
L1	3	0	
L2	7	3	
L3	5	6	
L4	14	8	
L5	0	2	
Thoracoplasty	Yes (24), No (5)	Yes (15), No (1)	0.399^†^
Intra-operative complications	0	0	
Neuro-monitoring signal change	0	0	
Screw pull out	0	0	
Immediate post-operative Cx.	3	2	0.991^†^
Infection	0	0	
Pulmonary complication (hemothorax or pneumonia etc.)	2	1	
Wound dehiscence	1	1	
Neurological deficit	0	0	
Abdominal discomfort	0	0	
Late complications	3	0	0.267^†^
Delayed infection	1	0	
Reduction loss	0	0	
Metal breakage	1	0	
Decompensation	1	0	
Internal organ complication	0	0	

*Notes*: Group A comprised patients treated with titanium-alloy rods and Group B comprised patients treated with cobalt-chrome-alloy rods. Flexibility of the major curve was calculated using the following formula: Cobb’s angle on standing view - Cobb’s angle on side bending view/Cobb’s angle on standing view × 100. BMI—body mass index, Cx - complications.

^*^Mann-Whitney U test, ^†^Fisher’s exact test. Significant differences are accepted for *P* value < 0.05.

The change in Cobb’s angle of thoracic curvewas 41.5 ± 13.0 degrees in the group A and 43 ± 12.2 degrees in the Group B. The thoracic curve correction rate was determined using the formula “preoperative major curve (°) - postoperative major curve (°)/preoperative major curve (°) × 100%”. It was 71.2 ± 6.8% in the group A and 71.2 ± 10.0% in the Group B. Similarly, the lumbar curve correction rate was 73.3 ± 13.6% in the Group A and 66.8 ± 13.1% in the Group B. There was no statistical difference in the thoracic or lumbar curve correction rates between the Ti and CCM groups (*P* = 0.664, 0.09).

At last follow-up radiography, maintenance of corrected Cobb’s angle for thoracic and lumbar curve was evaluated between Group A and B and there was no statistical difference between immediate and last follow-up corrected Cobb’s angle for thoracic and lumbar curve in Group A and B (*P* = 0.711, 0.722 in Group A, *P* = 0.271, 0.662 in Group B).

**Figure 1:**
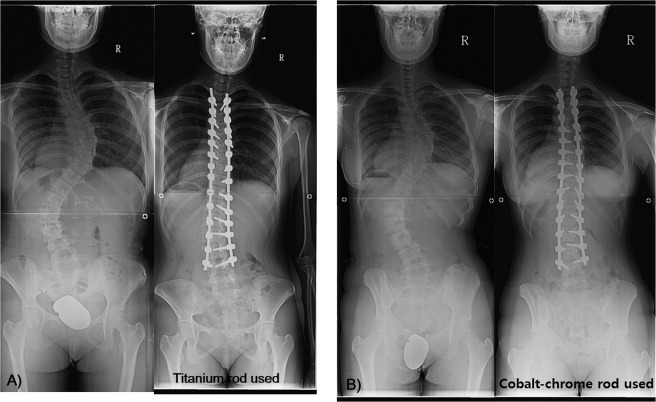
Comparison of coronal profiles between titanium and cobalt-chrome-alloy rods. (**A**) A 13-year-old girl with progressive scoliosis. The curve pattern was King type 1 and the Cobb’s angle was measured at 60 degrees, Risser grade 4. The spinal deformity was corrected with the rod rotation technique using Ti rods to 13 degrees (78% correction rate). (**B**) A 14-year-old girl with progressive scoliosis. The curve pattern was King type 1 and the Cobb’s angle was measured as 59 degrees, Risser grade 4. The spinal deformity was corrected with the rod rotation technique using CCM rods to 15 degrees (75% correction rate).

There were also no significant differences in pre- and postoperative T1 and CA angles between the two groups (Table 3). The CB correction rate after surgery was 1.5 ± 13.5 mm in the Group A and 4.7 ± 12.8 mm in the Group B and the difference between groups was not statistically significant (*P* = 0.441) (Fig. 1).

**Table 3:** Preoperative and postoperative coronal balance evaluations.

Factor	Group A (n = 29) Titanium-alloy rod	Group B (n = 16) Cobalt-chrome-alloy rod	p*
**Pre-operative Cobb’s angle**			
Thoracic curve	60.1 ± 14.2°	60.1 ± 12.3°	0.729
Lumbar curve	48.3 ± 12.6°	47.9 ± 12.6°	0.969
**Post-operative Cobb’s angle**			
Thoracic curve	16.4 ± 5.4°	17.0 ± 5.5°	0.756
Lumbar curve	12.9 ± 7.0°	15.8 ± 7.2°	0.245
**Cobbs’ angle at final follow-up**			
Thoracic curve	16.9 ± 5.0	18.8 ± 4.8	0.208
Lumbar curve	13.7 ± 7.0	16.8 ± 6.4	0.146
Preoperative clavicle angle	0.5 ± 3.5°	0.9 ± 2.2°	0.205
Postoperative clavicle angle	3.7 ± 3.1°	3.1 ± 1.6°	0.241
Pre-operative T1 tilt angle	1.4 ± 8.4°	8.1 ± 4.7°	0.198
Post-operative T1 tilt angle	5.3 ± 7.4	5.6 ± 6.0°	0.197

*Mann-Whitney U test. Significant differences are accepted for *P* value < 0.05.

**Figure 2:**
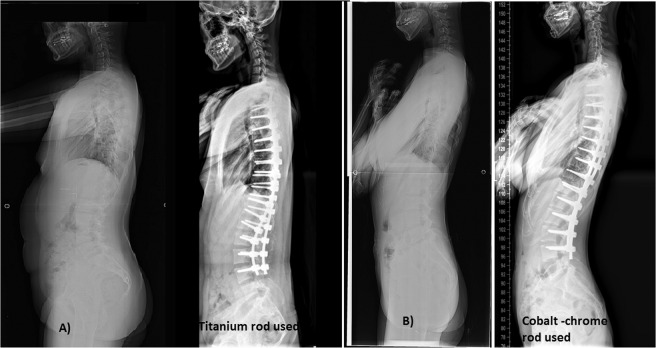
Comparison of the sagittal profiles between titanium and cobalt-chrome-alloy rods. (**A**) A 13-year-old girl with progressive scoliosis with a preoperative kyphosis angle of 15° and postoperative kyphosis angle of 19°. The lumbar alignment was 49° preoperative and 46° postoperative. (**B**) A 14-year-old girl with progressive scoliosis with a preoperative kyphosis angle of 12° and a postoperative kyphosis angle of 20°. The lumbar alignment was 45° preoperative and 51° postoperative.

Sagittal parameters between the groups were also similar, with no significant differences in SVA or TK (*p* = 0.485 for SVA, *P* = 0.086 for TK) (Table 4) (Fig. 2). However, the power for statistical results of TK is relatively weak, therefore, patients with CCM rods had greater postoperative lumbar lordosis (59.0 ± 3.6°) than patients with Ti rods (47.3 ± 9.1°) (*P* < 0.001). Postoperative TK in CCM rod group is larger than Ti rod group.

**Table 4:** Preoperative and postoperative sagittal balance evaluations.

Factors	Group A (n = 29) Titanium-alloy rod	Group B (n = 16) Cobalt-chrome-alloy rod	p*
Preoperative thoracic kyphosis	30.1 ± 15.9°	31.9 ± 13.8°	0.549
Postoperative thoracic kyphosis	26.4 ± 7.8°	30.6 ± 10.4°	0.086 (Power: 0.30)^¥^
Thoracic kyphosis at final follow-up	27.2 ± 7.3°	31.3 ± 10.2°	0.119
Preoperative lumbar lordosis	51.2 ± 13.9°	56.5 ± 11.1°	0.121
Postoperative lumbar lordosis	47.3 ± 9.1°	59.0 ± 3.6°	<0.001 (Power: 0.98)^¥^
Lumbar lordosis at final follow-up	49.4 ± 8.4°	58.4 ± 4.0°	<0.001
Preoperative spinal vertical axis	17.8 ± 24.0 mm	18.0 ± 12.7 mm	0.930
Postoperative spinal vertical axis	17.1 ± 33.4 mm	21.4 ± 18.8 mm	0.485

*Mann-Whitney U test. Significant differences are accepted for *P* value < 0.05.

^¥^For verifying the power of statistical results, G-power program (ver 3.19) was used.

At last follow-up radiography, maintenance of corrected TK and LL were evaluated between Group A and B and there were no statistical difference between immediate and last follow-up corrected TK and LL in Group A and B (TK: P = 0.691 in Group A, P = 0.852 in Group B, LL: P = 0.351 in Group A, P = 0.652 in Group B).

## Discussion

The rod-derotation maneuver after pedicle-screw insertion is a commonly used technique for curve correction in patients with AIS^1,2,4^. Mechanism of correction of rod-derotation is reducing the deformity by delivering the corrective force generated by derotation of rod anchored on screws to vertebrae. Rod-derotation maneuver is consisted of as follows; rod is pre-contoured fitting to the shape of scoliotic curve then pre-contoured rods are assembled to screws in concave and convex side and both rods are derotated 90 degree in counter clock wise direction using 3~4 vice grip.

For effective correction shape of pre-contoured rod should be maintained without deformation of flattening during derotation maneuver. Therefore, stiffer the rod is advantageous in maintain the pre-contoured rod shape. For these reasons cobalt-chrome-alloy which is approximately five times stiffer than titanium alloy is preferred by some surgeons. But not always cobalt-chrome-alloy bears higher correction rates due to pull out of screws from vertebra during rod derotation maneuver owing to too much stiffness of cobalt-chrome-alloy. On the other hand, even though titanium alloy is less stiff than CCM, Ti alloy has lower incidence of screw pull out due to its relative flexibility thus fulfilling its goal of correction without complications. Many studies compared effect of Cobalt-Chrome-alloy and Ti on correction rates in AIS surgery^16–24^. Etemadifar *et al*.^20^ reported that cobalt chromium-titanium (Co-Ti) rods were more effective than Ti only rods. However, their study used polyaxial pedicle screws which is less effective in transferring correction force to vertebral body due to freedom of head to body, and there was mixed use of relatively small-sized (5.5 mm) cobalt chromium and titanium on concave and convex side each. Considering these limitations, strong scientific validity is weak. Lamartina *et al*.^5^ reported better correction rates with a hybrid system using thicker rods (6 mm vs. 5.55 mm). However, this research did not exclusively studied pedicle screw constructs and also was not supported by an appropriate statistical analysis. Serhan *et al*.^23^ reported rod made of CCM and stainless steel materials are more corrective than that of titanium *in-vitro* study real scoliosis surgical conditions.

To overcome these limitations, this study was designed as prospective, consecutive, non-randomized method even though it was retrospectively analyzed. Double curve (Lenke type 6) only requiring fusion was enrolled and other variable mixed type of curves were excluded to avoid bias of patient selection. And for determination of distal fusion level (distal instrumented vertebra), Suk’s guidelines was used^6,12^. To the best of our knowledge, there are no papers that studied effect of material properties of the rod on correction rate with homogeneity of groups (e.g. single surgeon operation, single type of implant fixation, single pattern of curve).

With this background, study was carried out under the hypothesis that CCM (rigidity: 13.4 × 10^3^ ksi) rod is stiffer than Ti (rigidity: 5.90 × 10^3^ ksi) rod and thus can achieve better correction of scoliotic curvature. However, in contrary to initial assumption results showed no statistical difference in coronal or sagittal spinal parameters except for lumbar lordosis (Tables 3 and 4). Also this results contradicted the findings of a previously published study by Lamerain *et al*.^19^ that resulted as CCM rods produced higher correction rates in the frontal plane when compared to stainless steel rods of the same diameter (53.3% and 51.4%). But in that study patients operated during learning curve periods were not excluded thus showing higher correction rates among CCM rods group of patients operated after learning periods. Also, the study population was heterogeneous with inclusion of all types of curves in Lenke classification.

Our study is not the first study to show discrepancy between correction rates and rod stiffness of rods. The similar correction rates between stiffer and less stiff rods in our study can be explained by understanding that correctional force of rod-derotation is delivered to deformed vertebra via screws inserted in vertebra through pedicle. For the force of rod-derotation to be transferred to vertebra without loss of force, purchase of screws should be strong enough to resist to pulling out force of screws generated by derotation of rod. But in biologic conditions rod-derotation mechanical force exceeds purchasing power of screws resulting in pulling out or loosening of screws. Pulling out screws from vertebra fail to deliver the correction force effectively and results in loss of reduction sometimes. Ti rod although is less stiff than CCM rod, Ti rod is far stronger than the purchasing power of screws and thus Ti rod can achieve the effective correction.

In this study, sagittal parameter of lumbar spine (LL) definitively was affected by material property of rod (*P* < 0.001). And sagittal parameter of thoracic spine (thoracic kyphosis) also was affected by material property of rod in CCM-rod used group, even though it was not statistically proved (*P* = 0.086). Statistical results of TK had relatively weak value of power analysis (0.30) by small enrolled patients and /or absence of more definitive difference in measured thoracic kyphosis between two groups.

Abul-Kasim *et al*.^21^ also reported that larger rod diameter (stiffer rods) had a positive impact on the deformity correction in the sagittal plane with pre-operative hypokyphotic thoracic spine and mainly Lenke type 1 curve. In this study, sagittal profile in Lenke type 6 curve is also more affected by more rigid rod. These results could be explained as follows; firstly, various surgical techniques making rigid curve flexible mobile, multiple facetectomies to enhance efficient load transfer of mono-axial pedicle screw into vertebrae. Through these techniques, more powerful correction force of stiffer rod (CCM) can be transferred to vertebral body without pulling out of screws; Secondly, sagittal profile in lumbar and thoracic are compensatory working in interactively influencing with each other. The lumbar spine is relatively more flexible than thoracic spine. Hence when pre-contoured rods are maneuvered for deformity correction, the stiffer CCM rods would have lesser deformation in the thoracolumbar or lumbar region compared to Ti rods. This may be the cause of the greater postoperative lumbar lordosis observed with CCM rods and this large change of lumbar curvature also have effect on postoperative TK sequentially. In this study, postoperative TK in CCM-rod used group is larger than Ti-rod used group. However, the degree of change of postoperative TK is less than lumbar curvature. And the possible causes could be inferred to interactive action of thorax and lumbar curve each other and relative stiffness of thoracic spine by holding effect of rib cage.

In the mid-term follow-up for more than 5 years, it was confirmed that the coronal and sagittal balance was maintained regardless of the material properties of the rods. There were three delayed complications occurred in the Ti rod group and revision surgery was performed in infected case only. There was no statistical difference between the two groups (*P* = 0.267).

Our study was not without limitations. The study was not randomized and the number of enrolled patients was relatively small. Although this study was designed as a retrospective type, the data included in the study were collected and managed in a prospectively, consecutive, non-randomized method. Furthermore, to decrease the selection bias, titanium and cobalt-chrome rod were used to patient with time sequential pattern using all or none way. And to improve the quality of results in this study, we tried to apply to proper statistical analysis and verified power of statistical results, although achieving homogeneity of the enrolled patients in terms of a single curve was not possible. A large randomized study is required to provide more reliable information about the effect of rod stiffness on correction rates in patients with AIS. Various factors affect the outcome of such surgeries, such as the screw-bone interface, screw-rod interface, rod contouring and surgeon familiarity with the instruments. In spite of our efforts, these factors could not be controlled for in our study. However, we feel that our study is relevant given the fact that optimal rod stiffness to correct scoliotic curves has not yet been determined. In addition, the authors did not evaluate the clinical results before and after surgery. Clinical outcomes are not simply correlated to the radiological corrections but can be affected by changes in coronal and sagittal profile after operation. If the clinical outcomes were evaluated with health-related and functional outcomes questionnaire like SRS-22, the effect of rod material on the patients could be evaluated more carefully in terms of physical, mental and surgical satisfaction.

## Conclusions

Ti and CCM rods showed similar correction rates in the sagittal and coronal planes for the surgical correction of AIS with double major curves. Biomechanical studies of Ti and CCM rods *in vitro* is different in biological condition.
